# Knowledge Level of ICU Nurses Regarding Nutritional Assessment of Critically Ill Patients: A Systematic Review

**DOI:** 10.3390/nursrep14010045

**Published:** 2024-03-08

**Authors:** Vicente Doménech Briz, Vicente Gea-Caballero, Elena Chover-Sierra, Michal Czapla, Piotr Fehler, Antonio Rodríguez-Calvo, María Luisa Ballestar-Tarín, Patricia Marín-Maicas, Ana Cristina Cabellos-García, Marta Pardo-Bosch, Raúl Juárez-Vela, Antonio Martínez-Sabater

**Affiliations:** 1 Epidemiology Unit, Xativa Public Health Center, 46800 Valencia, Spain; vicendobri@gmail.com; 2 Faculty of Health Sciences, Valencian International University, 46002 Valencia, Spain; pmarinm@universidadviu.com (P.M.-M.); accabellos@universidadviu.com (A.C.C.-G.); 3 Research Group Community Health and Care, Salcom, Valencian International University, 46002 Valencia, Spain; 4 Facultat d’Infermeria i Podologia, Nursing Department, Nursing Care and Education Research Group (GRIECE) GIUV2019-456, 46001 Valencia, Spain; elena.chover@uv.es (E.C.-S.); m.luisa.ballesterr@uv.es (M.L.B.-T.); antonio.martinez-sabater@uv.es (A.M.-S.); 5 Internal Medicine Department, General Hospital of Valencia, 46010 Valencia, Spain; 6 Department of Emergency Medical Service, Wroclaw Medical University, 51-616 Wroclaw, Poland; michal.czapla@umed.wroc.pl; 7 Group of Research in Care (GRUPAC), Faculty of Health Sciences, University of La Rioja, 26006 Logroño, Spain; 8 Department of Anaesthesiology and Intensive Care, University Hospital, 50-556 Wrocław, Poland; 9 Department of Anaesthesiology, Salamanca Hospital Complex, 37008 Salamanca, Spain; arodriguezc@saludcastillayleon.es; 10 Hospital de la Ribera, 46600 Alzira, Spain; martapardob@gmail.com; 11 Care Research Group (INCLIVA), Hospital Clínico Universitario de Valencia, Universitat de Valencia, 46010 Valencia, Spain

**Keywords:** nutritional assessment, nutritional risk, nutritional status, intensive care unit, nursing

## Abstract

Background: Nutritional assessment on admission of critical patients is of vital importance to determine critical patients in whom there is a risk of malnutrition. Currently, it has been detected in most of the patients admitted to the Intensive Care Unit (ICU) that 60% of the daily calories are not achieved. Nurses play an essential role in the comprehensive assessment of the patient, including the nutritional area; however, significant deficits have been detected in some knowledge regarding Enteral Nutrition (EN). Objective: We aim to determine the level of knowledge of nurses in the nutritional assessment of critically ill patients. Methodology: A systematic review of the scientific literature was conducted using the PRISMA statement. Between January 2017 and February 2023, articles were rescued from the electronic databases “Pubmed”, “Scopus” and “The Cochrane Library”, which analyzed the level of knowledge of ICU nurses regarding nutritional assessment. Results: Most of the results found showed that nurses had deficient levels of knowledge in relation to nutritional assessment and practices. Interventions related to nutritional assessment were scarce, in contrast to those associated with the management of Nasogastric Tube (NGT) or patient positioning. Conclusions: The level of knowledge described was low or inadequate in relation to the care associated with the nutritional assessment of critically ill patients. The use of scales to assess the risk of malnutrition was not reported. This study was prospectively registered at PROSPERO on 25/10/2023 (insert date) with registration number CRD: 42023426924.

## 1. Introduction

We can understand ICUs (intensive care units) as small organizations in which correct knowledge management is crucial to generating beneficial results [1]. In the process of knowledge acquisition, first we find data, then information (interpretation of data), and then knowledge [1,2,3]. In addition, the knowledge required for nursing practice is as complex as the nature of nursing due to the social relationships derived from the care process and the health/illness binomial [4].

The levels of malnutrition reached in patients admitted to the ICU can be as high as 40–50% [5]. In addition, malnutrition associated with the lack of oral intake of the patient produces alterations in the structure and function of the intestinal mucosa, increased inflammatory response to the disease and infectious morbidity [5,6]. On the other hand, different studies have shown that in the majority of patients admitted to the ICU, not even 60% of the prescribed daily calories are provided [7,8]. These processes are related to severe catabolism caused by stress and proinflammatory cytokines [9,10,11]. Together with this fact, different studies indicate that it may be related to a deficit in nutritional assessment and delay in the establishment of Enteral Nutrition (EN) [6,8,12]. This malnutrition causes an increased length of stay in the ICU (an average of 5.6 to 6.6 days longer hospitalization), higher prevalence of nosocomial diseases, more frequent infections, longer duration of mechanical ventilation, higher mortality (up to three times the relative risk of death among patients with malnutrition at one and two years after discharge) or worse long-term recovery outcomes [13,14]. These negative consequences result in increased hospitalization costs, up to an average of more than EUR 8000 for patients at high risk of malnutrition [15,16,17].

Reductions in mortality after 90-day nutritional therapy have been reported in up to 51% of patients and reductions in readmission rates [8,14,18], probably due to nutritional therapy and EN being instituted after appropriate assessments in critically ill patients. Nutritional assessment on admission of critically ill patients is particularly important to determine those at risk of malnutrition, and nurses play a key role in nutritional assessment, as well as in the administration and ongoing care of EN. For effective administration of NE, the healthcare professionals responsible for administering this care must have sufficient knowledge as well as clear responsibilities (lack of responsibility for nutritional therapy often leads to inadequate nutritional outcomes) [19,20]. Regarding the nutritional knowledge of intensivists, a lack of leadership and application of the new nutritional guidelines in the management of critically ill patients has been observed in the scientific literature [8,10,12]. Furthermore, studies indicate a clear improvement in communication, knowledge and nutritional approach by intensivists after receiving nutritional programs focused on critically ill patients [10,13]. Significant deficits have been detected in some knowledge and training of nursing staff in relation to EN, as well as different perceptions associated with the nutritional assessment of critically ill patients, which leads to iatrogenic malnutrition and may compromise patient care. Although nurses generally showed a positive attitude towards the nutritional care of their patients, they had limited knowledge of nutritional assessment and detection of patients at risk of malnutrition. Barriers delaying the administration of EN were also reported due to different perceptions of nutritional care, lack of priority and lack of accountability [17,20]. Other studies showed that nurses presented a higher level of knowledge for advanced phases of the nutritional process, such as “prevention of complications”, as opposed to processes related to “assessment” or “identification of goals” [17,19,20]. These findings have presented a series of negative consequences described in the scientific literature: undernutrition, energy deficit, lower total nutrition for critically ill patients or higher mortality [7,10,17].

The ASPEN and ESPEN guidelines point out the need for interdisciplinary cooperation in the assessment and monitoring of the nutritional needs of critically ill patients since the main objective is to provide energy support to the patient due to their state of stress, to reduce the occurrence of possible complications due to malnutrition and its early onset, with the ultimate aim of improving their evolution [9,18,21,22]. One of the important practical recommendations is the need for evidence-based guidelines, a multidisciplinary approach to EN care and the formulation of nutritional support teams in critical care units. Also important is the improvement of nurses’ accessibility to sources of NE knowledge, such as scientific journal articles and international guidelines, along with refresher programs that improve the sense of responsibility among nurses [10,23,24].

In recent years, there has been an important development in nursing, particularly in the field of critical care, where new professional competencies have had to be acquired to improve the care of these patients [6,12,18,24,25]. The improvement in nurses’ training and professional environments implies better results in complex patients (reduction of nosocomial infections, lower prevalence of pressure ulcers, better follow-up of diseases, lower mortality rate) and a reduction in associated costs [26,27,28], which is why it is so important to evaluate nurses’ knowledge in these units and how to improve it, through integration in standardized, and at the same time individualized, care plans [25,27]. These positive results generate an increase in the quality of care, an objective pursued by any hospital center [29,30].

Thus, the research question that arises from this systematic review is: is the level of knowledge that nurses have regarding the nutritional assessment of critically ill patients sufficient?

The objective of our study is to determine the level of knowledge of nurses in the nutritional assessment of critically ill patients. As secondary objectives, we intend to describe the scales or questionnaires used to measure the level of knowledge, as well as the implications derived for the health of the patients and for the health organization of the nutritional assessment conducted by ICU nurses.

## 2. Materials and Methods

### 2.1. Research Design

Systematic review of the scientific literature carried out in the year 2023, using the PRISMA (Preferred Reporting Items for Systematic Reviews and Meta-Analyses) 2020 statement [31].

### 2.2. Search Strategy

The information retrieved for the review was from the last 6 years (1 January 2017 and 1 February 2023). A search in the electronic databases “Pubmed”, “Scopus”, “CINAHL” and “The Cochrane Library” was considered. The free and “Mesh” terms used were: “nutrition assessment”, “nutritional status”, “nutrition risk” “knowledge”, “hospital”, “care management”, “intensive care unit”, “perception”, “nurse”. The search was limited to articles found in English, Spanish or Portuguese. The bibliographic references of the retrieved articles were examined with the aim of finding other relevant articles (reverse search).

The selected articles were grouped according to the type of study and study variables (importance of the performance of nutritional assessment by nurses, nurses’ knowledge and perception of nutritional assessment, questionnaires that have been used).

The bibliographic manager “Mendeley Reference Manager” [32] was used to manage the retrieved documents. The selection of articles was carried out independently by two researchers.

The following table (Table 1) shows the search strategy used to retrieve the eligible documents in this systematic review, as well as the terms used in each database, the search period selected and the articles obtained.

### 2.3. Selection Criteria

Inclusion criteria: Studies that contemplate the level of knowledge that ICU nurses have regarding the nutritional assessment of critically ill patients and the repercussions of this assessment on the patient. Studies carried out in elderly patients in ICUs. Research carried out on patients over 18 years of age has been taken into account because, in this context, the nutritional risk scales adopt the same items, with greater capacity to compare the results with each other and be able to extract the knowledge nurses have about this type of scale. Types of studies: systematic reviews, randomized controlled trials and other experimental studies, observational studies and cross-sectional studies. 

Exclusion criteria: studies on pediatric patients or those belonging to other hospitalization units. Studies focused on pharmaceutical properties of EN or PN or those that do not determine the level of knowledge of nurses regarding nutritional assessment or do not analyze how this may impact the critically ill patient. Studies with a sample of pediatric patients have been discarded because the scientific literature shows a greater standard use of nutritional risk scales in adult critical patients, so it is more convenient for our research to evaluate nurses’ knowledge about nutritional risks in this type of patient.

### 2.4. Measures of Effect

The evaluation of the methodological quality was carried out in two phases: first, the evaluation/critical reading of each document and, subsequently, the verification of the level of bias.

For the quality assessment, the scale adjusted to the corresponding design was used: STROBE (Strengthening The Reporting of Observational studies in Epidemiology) [33], CASPe (Critical Appraisal Skills Programme) critical reading [34] and the ICROMS tool (Integrated quality Criteria for the Review Of Multiple Study designs) for mixed studies [35]. The cross-sectional studies were also evaluated using the instrument developed by Berra et al. [36].

Regarding the assessment of risk of bias, the NOS (Newcastle–Ottawa) scale was used for longitudinal non-randomized studies [37,38,39,40] and the ROBINS-I (Risk of Bias In Non-randomized Studies of Interventions) scale for quasi-experimental studies [41]. The latter instrument is recommended by the Cochrane Collaboration [42]. For the studies evaluated using the NOS scale, those with scores of less than 7 points were defined as having a high level of bias [43].

Finally, the Scottish Intercollegiate Guidelines Network (SIGN) tool [44] was used to evaluate and classify the studies according to the level of evidence.

### 2.5. Data Extraction (Selection and Coding)

The selection of documents was made first by title and then by reading the abstract. The selection was made by two independent investigators to identify studies that potentially met the inclusion criteria described above. For potentially eligible studies, the full text was retrieved and also assessed by both reviewers for eligibility. A third investigator served as reviewer in the case of discrepancy between the previous two.

For each study, data were recorded on a digital form, collecting the characteristics of the study (population, study design) and the main topic of the study related to one of the research variables (scales or questionnaires to measure knowledge, nurses’ perceptions).

### 2.6. Data Synthesis Strategy

A formal narrative synthesis was made of the findings of the included studies, classifying the results according to the role of nurses during the nutritional assessment of critically ill patients, the nurses’ knowledge, perceptions and attitudes regarding EN and the scales or questionnaires used. The scales or questionnaires used refer to those used by the researchers in each of the studies.

## 3. Results

**Figure 1:**
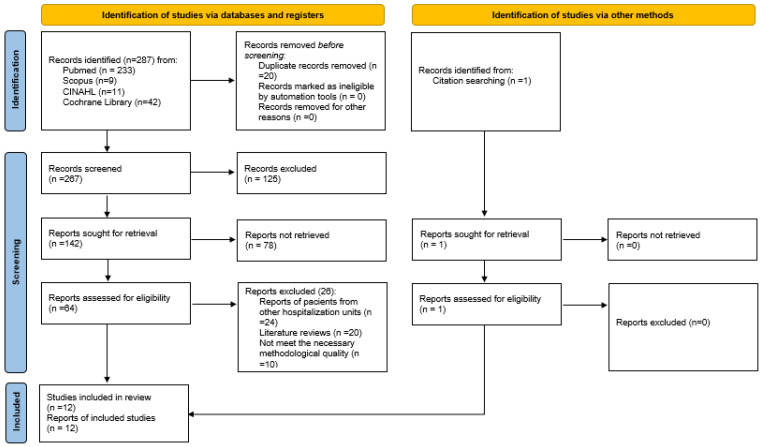
Flow chart (Page 2021) [31]).

The initial search yielded a total of 287 articles, of which 11 were finally selected, to which we added 1 article found through a reverse search, so that 12 articles were finally obtained for the systematic review. The selection process is shown in Figure 1.

In terms of study design, eight cross-sectional descriptive studies [45,46,47,48,49,50,51,52], two quasi-experimental studies [53,54], one literature review [55] and one mixed study [56] were collected. By origin, two were from the United States [54,55], two from Australia [45,56], one from Yemen [50], one from Ethiopia [49], one from Palestine [47], one from Israel [51], one from South Korea [53], one from Greece [52], one from China [48] and one from Jordan [46].

Table 2 and Table 3 show a summary of the selected articles, taking into account the variables of interest.

### 3.1. Evaluation of the Level of Bias

The quasi-experimental studies [53,54] and the retrospective study [45] have been classified as having a low risk of bias because they had a more robust design with a greater capacity to control errors [38,39,40,41].

The cross-sectional studies and the literature review have been considered of moderate risk [36,38,57] since the study designs themselves entail the existence of associated biases. However, we believe it is appropriate to include them in the present review because they have provided useful information to estimate the frequency of nurses with high or low levels of knowledge about EN or the possible association between this knowledge and the health status of patients admitted to the ICU, as well as the description of nurses’ perceptions, barriers or attitudes [36,57]. We also double-checked the quality of the cross-sectional studies using the instrument developed by Berra et al. [36] in order to include those studies with higher scores.

### 3.2. Nurses’ Knowledge and Perceptions of En

The study by Hadera, Worku and Tuli [49] described that almost two-thirds of the surveyed nurses (67.7%) had insufficient knowledge, while only 32.3% of the nurses had acceptable knowledge about NE. The results [47,49,50,51,53] showed a mean score of insufficient knowledge, in some cases 9.6 ± 2.8 out of 20 (describing it as inadequate) [47]. Meanwhile, 54.8% of the nurses responded that they were unaware of the guidelines on EN in their ICU, and 60.4% stated that there were no procedures on nutrition in their workplace [47].

According to the results, the factors most associated with patient undernutrition were lack of resources, lack of knowledge and work overload [46,48,51]. Other related factors were physical exhaustion, stress, understaffing and lack of incentives [46,47,48,49,56]. This underfeeding was also related to the early initiation of EN [45] and the inability of nurses to rapidly initiate EN pre-prescription volumes, which negatively impacts patients (in their study, only 9.3% of patients initiated EN at 12 h; of these, only 71.4% met nutritional requirements at 72 h after admission, and these types of patients were more likely to survive). Other studies also described a delay in achieving EN flow [48,52,56], as 83.1% of the nurses interviewed reported that this flow was determined by medical orders [52], despite the fact that most respondents stated that EN was extremely important and should be initiated as soon as possible [48,56]. In addition, it was noted that EN-related nursing care was consistently documented when associated with other patient priorities, with EN being given higher priority in more stable patients [56]. Surveys conducted by several authors [46,48,54] (prior to the implementation of the contemplated nutrition education program) found that the main individual and team barriers to initiating optimal EN were feeding intolerance, standard nursing practices, severity of illness, lack of knowledge and beliefs that new guidelines would not change current practices. After the educational interventions were implemented [54], the level of knowledge of professionals regarding EN improved by up to 81%, interdisciplinary communication increased and feeding initiation times and complications were reduced [53,54]. In addition, they noted that positive attitudes toward a change in current guidelines led to a better success rate of the educational program [47,52,54].

However, we obtained two contrasting results to the previous data, in which nurses showed a high level of knowledge regarding EN-related practices, and most of the nurses’ practices were in accordance with international guidelines, such as back elevation and catheter flushing [48,52]. However, it was also shown that there were significant differences between the knowledge they applied in practice and the guidelines or protocols they should follow. Similarly, 81.4% of the nurses were aware of the guidelines on EN, and 65.2% stated that a protocol existed in the ICU [52], although it was not applied in clinical practice.

It was also visualized that nurses’ education was strongly related to EN administration [48,51,52,53], and those nurses who had received EN training were twice as likely to perform better nutritional practices than those who had not [49,52]. Furthermore, the greater the nurses’ knowledge, the more they conformed to current nutritional guidelines, with a moderately strong correlation [46,51,53]. The implementation of a protocol reduced the time to initiation of EN and a higher rate of compliance with the caloric and nutritional target (several studies indicate increases of up to 80% of patients who have reached a caloric target after implementing an EN protocol) [55], coinciding with previous findings, i.e., greater knowledge and training in the area of EN allowed standardization of practice and better patient care. Experience was also related to lower barrier scores, and those nurses with more experience became training resources and information channels [48]. In addition, when nurses lack knowledge of EN, the presence of nutritionists in the ICU is not properly utilized, which significantly interferes with feeding management [48].

Among the areas of knowledge deficits, it was noted that half of the nurses did not know the differences between enteral products, complications, when the position of the bed could be changed, administration of drugs or contraindications of EN in immunosuppressed patients [47,50]. Other areas where lack of clarity was described were the prevention of complications related to EN [46,51]. Similarly, it was found that 91.1% of nurses did not consider nutritional care and follow-up to be their responsibility and that in this area, it belonged to the dietician or physician [50,51,52].

### 3.3. Scales and Questionnaires Used

Several authors [50,53] used a self-administered questionnaire developed by Persenius et al. [58], modified it and obtained demographic data and nurses’ perceptions of EN (responsibility, knowledge, management support, sources of EN knowledge and opinion on the need for EN training). A Likert-type scale, ranging from 1 (process never performed) to 5 (process very frequently performed), was used to classify the scales [50,51,53] and ranging from 1 (process never performed) to 5 (process very frequently performed). Cronbach’s alpha coefficients for these types of perceptions had a mean of 0.8 [50] and 0.84 [53]. Other studies also conducted surveys similar [46,47,49,51,56] to the one mentioned above, which consisted of a questionnaire based on pre-interviews with nurses. Huang et al. [48] conducted two types of questionnaires, one on general demographics and how NE is managed and another on observed barriers to enteral feeding of critically ill patients [46,48,56] based on Cahill et al. [59]. Cronbach’s alpha was calculated for all subtypes of scales, and a high level of internal consistency was found for this scale (all values were between 0.66 and 0.85) [46]. 

A similar questionnaire was used by Dokoutsidou et al. [52], specifically obtained from the “Nursing practices of enteral nutrition Questionnaire” [60], which consisted of dichotomous or single-response questions and was structured in three sections: general practices in the ICU regarding EN (responsibility, patient position during administration, indications for delay in initiation, etc.), management of intolerance to EN (continuous assessment of tolerance, management of gastric residual volume, assessment of the same by aspiration and management of gastric contents) and management of complications (control of patency, obstruction and frequency of tube cleaning, assessment of pulmonary aspiration and management of diarrhea). Demographic and occupational data were also included in the same questionnaire.

Other authors [45] used a case report form in their retrospective study, which contained patient demographics, NE characteristics, time of onset, patients’ nutritional needs, documented nursing practices, causes of feeding delays and dietitian reviews.

Table 2 shows a summary of the items evaluated and the psychometric properties of the scales used by the different authors.

### 3.4. Nutritional Assessment of the Critically Ill Patient: Implications

The functions described in the results [45,46,47,48,52,53,55,56] have been more related to NGT (nasogastric tube), patient positioning, documentation of nutritional support, treatment and NE administration than to assessment on admission or risk factors of critically ill patients. It was also identified that these functions were recorded to a lesser extent during the beginning of the shift. After various educational interventions, activities associated with nutritional care improved significantly [53], as did the time to initiate enteral feeding, with a reduction from a mean of 53.6 h pre-intervention to a mean of 40.2 h post-intervention [54] (the improvements were statistically significant). In addition, 71% of respondents stated that nutrition education interventions improved communication, and 81% felt that these measures were helpful in initiating early NE. More than 80% of respondents claimed to have increased their knowledge of EN.

Other roles that nurses perceived as more of their own were daily monitoring of electrolyte levels and blood glucose or informing the physician of any abnormalities in blood work [45,51].

Several studies [45,51,52] described that dietitians or physicians performed better dietary follow-up. 

Nurses were recognized to play an important role in the implementation of a protocol for EN management [53,55], as they were often the first clinicians to assess nutritional status. They also sought to reduce unnecessary interruptions and achieve EN target rates, thus improving the care provided and avoiding adverse outcomes [53,54]. Nurses who are more committed to all nutritional interventions (assessment on admission, early initiation of EN, defense of EN, management of complications, etc.) generate better care in critically ill patients, which results in a decrease in hospital stay and comorbidities associated with the critical state [50,51,52,53,54].

## 4. Discussion

The aim of our study was to evaluate the levels of knowledge of ICU nurses with respect to nutritional assessment, the scales used and the implications for the health of patients carried out by ICU nurses. So far, the present review is the only one in the scientific literature that has taken into account the scales for the assessment of nurses’ knowledge and their possible comparison in order to detect common elements and aspects that could be improved, both related to the questionnaires themselves and to the nurses’ practices.

Most of the results found have shown that nurses present poor levels of knowledge in relation to nutritional assessment and practices [46,47,49,50,51,53], and the highest scores obtained were those associated with NGLS management, patient positioning or NE assessment [45,46,48,49,51,52,56,61], and not with nutritional assessment on admission. These findings are in line with the scientific literature [17,19,62], where it is shown that nurses consider nutritional care less important and do not take into account the application of scales or instruments to assess whether or not patients are at risk of malnutrition. It is true that some of our results [47,52] and other scientific articles [17,58] do show higher scores in nutritional care, but at the same time, they reported that nurses were not able to identify malnutrition in critically ill patients or that nutritional guidelines were not applied correctly. In addition, it was also detected that in many of the ICUs analyzed, there was no NE protocol [12,17,23,46,47,49,50,58,63], which prevents correct evidence-based practice and the achievement of optimal caloric-nutritional objectives for patients. The application of protocols based on current ASPEN or ESPEN nutritional guidelines [11,18,22] would allow a reduction in the time to initiation of EN and a higher rate of caloric compliance, and thus, an improvement in the health of critically ill patients.

The source of nurses’ knowledge about EN is also an important aspect, where the literature highlights that “Internet” is one of the major sources of knowledge, followed by “scientific courses”, “nursing education or school” and “consultation with peers” [12,17,19]. Some of our results indicate the same problem [46,47,48,50]. Some of the barriers to the underutilization of scientific journals were research quality, insufficient time, lack of interest or lack of knowledge [12,17,20]. These facts highlight the need to increase formal education on admission assessment and management of EN in order to reduce comorbidities and ICU stay.

The nurses also showed in the questionnaires that another of the missed barriers was the absence of nutritionists in their critical care units [48,50,53,55]. Nutritionists are considered key personnel to guide nutritional care, and in the absence of this personnel, nurses use other sources of knowledge, such as those reported previously [12,48,49,50]. Morphet et al. [17] reported in their study that one of the main sources of information and knowledge for nurses was nutritionist advice and protocols. Furthermore, scientific societies such as ASPEN or ESPEN [11,13,17,18] recommend the presence of a multidisciplinary team made up of doctors, nurses, nutritionists and other health professionals in order to offer the best nutritional care.

Regarding the scales to assess nurses’ knowledge, a great diversity has been found [45,46,47,48,49,50,51,52,53,54,55,56], so that all collected common questions, such as demographic data or nurses’ perceptions about EN (responsibility, sources of information or nutrition management), and some other studies also explored the barriers perceived by nurses [45,46,48,49]. Despite the fact that all scales presented a high level of internal consistency, with a mean score above 0.8, there is a need for greater homogenization of these instruments in order to be able to assess and compare with a better degree of confidence nurses’ perceptions of EN and their knowledge [48,51,55].

The importance of evaluating the level of knowledge of other frequent pathologies in the ICU, such as pneumonia associated with mechanical ventilation, pain care and pressure ulcers (PU) or endotracheal aspiration [64,65,66,67], has also been observed in the scientific literature, demonstrating that the determination of the level of knowledge is crucial for maintaining optimal care and that this always improves with educational interventions.

The results of our study indicate that the nurses’ functions were more related to the onset of EN, its management and defense (such as early onset) [46,46,48,49,51,52,56,61], not showing an assessment at the admission of the nutritional status of the patients or using the tools proposed by ASPEN, ESPEN or scientific evidence [16,18,22,25,68]. These findings coincide with the scientific literature [19,63,65,68], where it is shown that nurses consider nutritional care less important and do not take into account the application of scales or instruments to assess whether or not patients are at risk of malnutrition. In addition, the highest knowledge scores were associated with the items on the implementation of interventions (initiation of EN, management of EN), followed by the prevention of complications. The lowest scores were for outcome evaluation [45,50,52]. The higher performance of functions related to NGLS placement, glycemic control or informing the physician of the patients’ situation was also reported [45,49,54]. The ICU nurses interviewed have shown less perceived responsibility and “duty” for nutritional care, which may have a negative impact on the health status of critically ill patients [12,23,50,51,52,53,54].

The scientific literature [9,10,18,58,61,67] shows how nurses who are more committed to all nutritional interventions (assessment on admission, initiation of EN, management, management of complications, etc.) generate better care in critically ill patients, which has an impact on a decrease in hospital stay and comorbidities associated with the critical state. Educational interventions produce greater involvement and a source of knowledge suitable for nurses to develop their care and can act according to protocols in order to initiate EN as early as possible (usually nurses are the ones who experience the first contact with the patient) [48,55] and adverse outcomes can be avoided [18,53,62].

### 4.1. Limitations and Future Lineas of Research

This study has some limitations. We are aware that cross-sectional studies may have more types of biases, such as the risk of selective reporting of the analysis and generating low evidence, being one of the limitations of this study. Another limitation of this study is the heterogeneity of instruments found to assess nutritional knowledge since we have found several scales (see Table 2) with similar objectives but assessing different aspects. Therefore, studies with more robust designs, such as experimental designs, are recommended to verify the true extent of nurses’ level of knowledge of nutritional assessment in the health of critically ill patients, and since no results have been obtained from Europe, it is encouraged to contribute to the evidence in the European territorial framework.

### 4.2. Implications for the Practice

The results have reported that nurses have low levels of knowledge in relation to nutritional evaluation and practices. It has also been described how interventions on nursing staff and, in general, on healthcare personnel in critical care units have had positive repercussions on their nutritional knowledge and make it possible for this type of care to be prioritized, being less perceived as important before interventions on professionals. A correct acquisition of nutritional knowledge and practices will result in an improvement in the health of critically ill patients, which is why education in this field of medicine is of vital importance to offer quality care.

## 5. Conclusions

The level of knowledge described in our results has been qualified as low or inadequate in relation to nutritional care associated with EN. Furthermore, no activities derived from the assessment of the nutritional status of critically ill patients on admission to the ICU or the use of scales to assess the risk of malnutrition were reported. It has been shown that those nurses with better knowledge scores or who had attended training on EN showed a better adaptation of nutritional practices to the current guidelines, thus demonstrating the importance of educational activities and training to improve the health of critically ill patients.

It is very useful to measure the level of knowledge and perceptions in relation to the nutritional practices of critically ill patients, to know in which competencies nurses can improve this knowledge and to what extent they can benefit from nutritional educational programs. In relation to the scales used to measure nutritional knowledge, a wide variety of instruments were used, sharing common elements such as demographic data, nurses’ perceptions and barriers encountered. It is important that the same scales were used in order to efficiently compare nurses’ knowledge and nutritional practices. In future studies, the same scales could be used to compare the results using the most common items observed in the present review, increasing scientific evidence on the true knowledge of nurses about nutritional care and what areas should be improved.

## Figures and Tables

**Table 1:** Search strategy.

Database	Search Strings	Retrieved Articles	Selected Articles
Pubmed	((Nutritional assessment) AND (knowledge)) AND (nurse)	109	7
	((nutritional assessment) AND (nurse) AND (knowledge)) AND (hospital)	71	0
	(((nutritional assessment) AND (intensive care unit)) AND (knowledge)) NOT (pediatrics)	11	0
	(((nutritional assessment) AND (perception)) AND (intensive care units)) NOT (pediatrics)	42	0
Scopus	(nutritional assessment) AND (intensive care unit) AND (knowledge) AND (nurse)	8	4
	(nutritional status) AND (intensive care unit) AND (knowledge) AND (nurse) AND (perception)	1	0
Cinahl	(nutritional assessment) AND (knowledge) AND (intensive care unit)	2	0
	(nutritional assessment) AND (intensive care unit) AND (knowledge) AND (nurse)	1	0
Cochrane Library	(nutrition assessment) AND (knowledge) AND (intensive care unit)	26	0
	(nutritional risk) AND (intensive care unit) AND (knowledge) NOT (pediatric)	16	0
Total Articles		287	11

**Table 2:** Results found (authors)—scales.

Items Evaluated	Alsa-Sayaghi et al., 2022 [50]	Darawad et al., 2018 [46]	Hamdan et al., 2022 [47]	Hadera et al., 2022 [49]	Ben and Theilla, 2021 [51]	Bloomer, Cake and Morphet, 2018 [56]	Dokoutsi-dou et al., 2021 [52]	Crossfield, Russo and Bucknall, 2021 [45]	Huang et al., 2018 [48]	Barhorst, Prior and Kanter, 2023 [54]	Jordan and Moore, 2019 [55]	Kim and Chang, 2018 [53]
Demographic data	X	X	X	X	X	X	X	X (of patients)	X			X
Knowledge of the responsibility for nutrition (existence of guidelines, who is responsible for NE, etc.).	X	X			X				X		X	X
Source of knowledge about EN (continuing education, conferences, articles, internet, etc.)	X		X						X			X
Responsibility (for assessment of nutritional status, implementation of interventions, etc.)	X	X	X		X	X	X		X	X	X	X
Knowledge (assessment of nutritional status, planning of interventions and goals, prevention of complications, etc.)	X		X	X	X	X	X		X	X	X	X
Documentation (support, existence of protocols, etc.) Enteral feeding interventions (tube flushing, cleaning after medication administration, etc.)	X	X		X				X	X		X	X
Enteral feeding interventions (flushing tube, cleaning after administering medication, etc.)	X	X	X	X	X	X	X	X	X	X	X	X
Potential resources (presence of dietician, interdisciplinary team, etc.)		X				X		X	X	X	X	X
Psychometric properties (reliability, validity)	X	X	X	X	X	X	X (only validity)	X (of patients)	X			X

**Table 3:** Summary table.

Authors Year Country	Type of Study Sample	Intervention Scale Used	Variables Results	Results	Conclusions	Assessment of Study/Risk of Bias	Level of Evidence: Sign
Barhorst, Prior and Kanter. 2023. United States. [54].	Quasi-experimental study. -20 patients admitted to a neurological ICU in the USA. -Implement a project of good practices related to NE and analyze it for 90 days.	-Implement a project of good practices related to EN and analyze it for 90 days. / -Check nutritional.	-Prevalence of nutritional risk. -Negative impact of delay in NE administration. -Attitudes of professionals towards the implementation of the project. Pre- and post-intervention level of knowledge.	-Application of clinical practice guidelines increased compliance with 48-h feeding by 100%. -Initiation of EN improved by 25%. -71% of respondents reported improved communication during EN implementation. The nursing-assessed checklist achieved 93% equality with the clinical practice guideline.	-Significant improvement in EN-related practices was demonstrated after implementation of the clinical practice guideline package. -An educational brochure and a package with all the necessary elements will be developed to ensure that the effect lasts over time.	ICROMS: 31/36 / ROBINS-I: Low risk of bias.	2+
Alsa-Sayaghi et al. 2022. Yemen [50].	Quantitative, descriptive, cross-sectional study. -292 critical care nurses.	-Investigated Yemeni critical care nurses’ perceptions of their responsibility and knowledge of enteral nutrition. / -Self-administered questionnaire.	-Demographic characteristics of study participants. -Nurses’ perceptions of responsibility and knowledge in enteral nutrition. Nurses’ sources of knowledge.	-Most nurses stated that there were no guidelines on EN in their units. -The internal conflict in Yemen influenced the implementation of guidelines. -Critical care nurses reported low responsibility and low knowledge about EN. -The main sources of knowledge were scientific conferences.	-The results revealed a lack of guidelines or protocols on EN in most critical care units. -Nurses perceived a low level of responsibility and knowledge and moderate support of documentation systems regarding EN.	STROBE: 22/22 Cross-sectional study: high quality. / NOS: 7/9	3
Hadera, Worku and Tuli. 2022. Ethiopia [49].	Cross-sectional observational study. -Surveys of 196 nurses working in public hospitals in Ethiopia.	-To assess the knowledge, practice and factors related to enteral nutrition in adult patients admitted to ICUs of public hospitals in Ethiopia. -Structured, self-administered questionnaire.	-Level of nurses’ knowledge. -Practices related to EN.	-67.7% of nurses presented inadequate knowledge and 53.8% of nurses reported poor practices related to EN. -Factors related to NE practices were age, NE training and availability of guidelines and protocols.	-A large proportion of nurses had inadequate NE knowledge and poor practices. -NE relied more on opinions than evidence-based methods.	STROBE: 21/22 Cross-sectional study: High quality. / NOS: 7/9	3
Hamdan et al. 2022. Palestine [47].	Cross-sectional observational study. -325 nurses who were working in 6 hospitals in Jordan.	-Investigate nurses’ knowledge, practices and attitudes regarding NE. / -Questionnaire	-Sociodemographic data of nurses. -Knowledge and attitudes towards EN.	-The mean knowledge score was 9.6 ± 2.8 out of 20. -Some practices showed differences with international guidelines. -Attitudes of nurses were positive towards EN.	-Nurses’ knowledge of EN was insufficient, which could increase the risk of mortality. -Nurses’ NE practices among nurses were inconsistent with current best evidence. -Nurses’ knowledge was influenced by nurses’ attitudes.	STROBE: 22/22 Cross-sectional study: High quality. / NOS: 7/9	3
Crossfield, Russo and Bucknall. 2021. Australia [45].	Retrospective study. -Retrospective review of medical records (150 eligible patients).	-Describe current practice on EN and identify barriers to optimal nutritional management in the ICU. / -Case report form.	-Sociodemographic characteristics of the nurses. -Initiation of EN and nutritional goals. -Barriers to optimal EN.	-Median time elapsed from patient admission to initiation of EN was 12.6 h, with 59.3% initiated within 12 h of admission. -16% of patients received 80% of nutritional goals within 72 h of admission. -Patients who received an initial dietary review within 24 h were more likely to achieve nutritional requirements.	-Airway management, procedural requirements, and delay in dietitian review for initiation of and delay in dietitian review for initiation of EN delivery to critically ill patients. -Addressing these barriers may require -Days one and two are problematic for NE administration.	STROBE: 20/22 / NOS: 8/9	2-
Ben and Theilla. 2021. Israel [51].	Cross-sectional descriptive study. -45 nurses working permanently in an ICU.	-To assess the roles of ICU nurses in the provision of nutritional care. / -Self-administered questionnaire.	-Sociodemographic data. -Nurses’ perception and knowledge of their role in nutritional care delivery.	-91.1% of nurses agreed that dietitians were the only ones who performed dietary follow-up. -75.6% of nurses reported that hypophosphatemia was rare in critically ill patients. -There was a statistically significant correlation between the nurses’ level of knowledge and their actual practice according to the guidelines; the higher the knowledge, the better the practices conformed to the nutritional guidelines.	-There is a need to improve practices related to the administration of nutritional care. -There is a significant relationship between nurses’ level of knowledge and better nutritional practices.	STROBE: 21/22 Cross-sectional study: high quality. / NOS: 7/9	3
Dokoutsidou et al. 2021. Greece [52].	Cross-sectional descriptive study. -70 ICU nurses from a tertiary hospital in Athens.	-To evaluate nursing practices with respect to l NE in the ICU. / -Nursing practices of enteral nutrition questionnaire.	-Sociodemographic characteristics of the nurses. -General practices. -Management of intolerance. -Complications	-Nurses who were aware of EN guidelines and who had attended training seminars reported correct answers more frequently regarding general practices, intolerance management and complications. -Among the nurses’ responses there were differences from theoretical knowledge to usual practice. -The increase in NE flow was mainly determined by physician orders.	-The implementation of continuing nursing education programs would improve nurses’ knowledge of EN and improve practices in relation to EN, leading to better clinical benefits. -The use of current literature would reduce knowledge gaps among nurses and increase participation in the multidisciplinary team in the ICU.	STROBE: 20/22 Cross-sectional study: High quality. / NOS: 7/9	3
Jordan and Moore. 2019. United States [55].	Bibliographic review. -10 articles obtained from the eligibility criteria.	-To determine the current status of evidence-based protocols for the administration of EN in critically ill patients.	-Time period to initiation of EN. -Caloric target -Target compliance rate -Complications -Role of nurses.	-All but one study picked up the importance of early NE initiation for maximum benefit. -Several studies demonstrated that use of an EN protocol improved practice standardization. -Nurse-led protocols for NE administration are recommended, as they are the first professionals to assess nutritional status.	-Interprofessional collaboration is paramount in order to achieve correct EN administration. -A nurse-led protocol may result in greater compliance and greater efficacy than a non-nurse-led protocol. -Current practice should be aligned with the best evidence.	CASPE: 8/10 / ROBIS: Moderate risk.	3
Kim and Chang. 2018. South Korea [53].	Quasi-experimental study. -205 critical care nurses from four hospitals in South Korea.	-Evaluate the effects of an educational program to improve critical care nurses’ perceptions, knowledge and practices regarding enteral nutrition. / -Data collection: questionnaire with four sections (perceptions, knowledge, practices and demographics). -Educational program: pre-interview and two lecture sessions.	-Demographic data. -Perceptions, knowledge and practices related to NE.	-Only 37% of nurses reported having had opportunities for nutrition education. -The main sources of participant knowledge were educational programs conducted at the hospital and peer consultation. -Scores related to perception, knowledge and practices related to NE improved significantly after receiving the educational program.	-The educational program employed improved nurses’ care of patients by providing more effective nutritional support.	ICROMS: 32/36 / ROBINS-I: Low risk of bias.	2+
Huang et al. 2018. China [48].	Descriptive, cross-sectional, multicenter study. -820 ICU nurses from 10 hospitals in China.	-Investigate the difficulties in administering EN to critically ill patients through the nursing perspective. / -Demographics questionnaire. Questionnaire on barriers to EN.	-Sociodemographic data. -NE delivery. -Critical care staff attitudes and behavior. -ICU resources.	-Delay of EN was the most frequently observed item. -Lack of professional knowledge is a factor that hinders the administration of EN. -When the nursing staff lacks knowledge of EN, the presence of nutritionists in the ICU is not taken advantage of.	-Factors that hindered NE administration were NE-related training, the presence of a nutritionist, hospital grade, specific protocols and professional qualifications.	STROBE:20/22 Cross-sectional study: high quality. / NOS: 7/9	3
Darawad et al. 2018. Jordan [46].	Cross-sectional descriptive study. -131 nurses from different hospitals and health sectors in Jordan.	-To explore the barriers perceived by Jordanian ICU nurses to NE. / -Self-administered questionnaire	-Sociodemographic data. -Barriers to EN (ICU resources, perceptions, protocols, attitudes).	-66% of respondents reported that they had never received any previous training on EN. -The most important barrier was related to the insufficient number of nurses, followed by fear of adverse events. The non-existence of a feeding protocol was also a significant barrier. -Nutritional care was generally considered a secondary priority.	-Participants focused more on insufficient ICU resources and availability of healthcare professionals. These barriers are modifiable, so identifying them is crucial for optimal patient care. -NE is a liability and delaying NE predisposes patients to malnutrition and undernutrition.	STROBE: 19/22 Cross-sectional study: high quality. / NOS: 7/9	3

## Data Availability

Under request on first author.

## Public Involvement Statement

No public involvement in any aspect of this research.

## Guidelines and Standards Statement

Systematic review of the scientific literature using the PRISMA (Preferred Reporting Items for Systematic Reviews and Meta-Analyses) 2020 statement [31].
